# Sex-Based Differences in Clinical Presentation, Management, and Outcomes in Patients Hospitalized with Pulmonary Embolism: A Retrospective Cohort Study

**DOI:** 10.3390/jcm14155287

**Published:** 2025-07-26

**Authors:** Benjamin Troxler, Maria Boesing, Cedrine Kueng, Fabienne Jaun, Joerg Daniel Leuppi, Giorgia Lüthi-Corridori

**Affiliations:** 1 University Institute of Internal Medicine, Cantonal Hospital Baselland, 4410 Liestal, Switzerland; benjamin.troxler@stud.unibas.ch (B.T.); cedrine.kueng@ksbl.ch (C.K.); fabienne.jaun@ksbl.ch (F.J.); joerg.leuppi@ksbl.ch (J.D.L.); 2 Faculty of Medicine, University of Basel, 4056 Basel, Switzerland; 3 Harvard T.H. Chan School of Public Health, ECPE, Boston, MA 02115, USA

**Keywords:** sex differences, pulmonary embolism, hospital management, retrospective study, acute care quality

## Abstract

**Background/Objectives**: Pulmonary embolism (PE) remains a major cause of morbidity and mortality. Despite advances in care, its nonspecific symptoms pose diagnostic and therapeutic challenges. Emerging evidence suggests sex-based differences in PE presentation, management, and outcomes, yet real-world data from European settings remain scarce. This study aimed to investigate sex differences in clinical presentation, diagnostic workup, therapeutic interventions, and outcomes among hospitalized PE patients. **Methods**: We conducted a retrospective cohort study including all adult patients (≥18 years) admitted with a main diagnosis of acute PE at the Cantonal Hospital Baselland between January 2018 and December 2020. Data were extracted from electronic medical records and included demographics, comorbidities, symptoms, diagnostics, treatments, and outcomes. Sex-based comparisons were performed using univariate analyses. **Results**: Among 197 patients, 54% were women. Compared to men, women were more often admitted by ambulance (42% n = 45 vs. 24% n = 22, *p* = 0.009), had more frequent tachycardia (38% n = 41 vs. 23% n = 21, *p* = 0.024), and received lysis therapy more often (10% n = 11 vs. 2% n = 2, *p* = 0.023). DVT was more frequently diagnosed in women when sonography was performed (82% n = 49 vs. 64% n = 34, *p* = 0.035). Men had higher rates of B symptoms, smoking, and family history of PE. Women had longer hospital stays and were more frequently discharged to rehabilitation facilities. No sex differences were found in in-hospital mortality, 6-month rehospitalization, or adherence to diagnostic guidelines. **Conclusions**: This study reveals sex-based differences in PE presentation and management, suggesting potential disparities in care pathways. Further research is needed to promote equitable, personalized treatment strategies.

## 1. Introduction

Pulmonary embolism (PE) remains a significant cause of morbidity and mortality worldwide, placing a considerable burden on emergency and inpatient care systems [1]. In Switzerland, the incidence rate of acute PE has increased notably, from 0.87 per 1000 population in 2003 to 1.19 per 1000 population in 2022 [2]. Despite advances in diagnostic tools and therapeutic strategies, PE continues to pose diagnostic and clinical challenges, particularly in the emergency setting where its symptoms can be nonspecific and easily mimic other cardiopulmonary conditions [3,4,5].

Increasing evidence suggests that sex-based disparities influence risk factors, clinical presentation, diagnostic pathways, treatment decisions, and outcomes [6,7,8,9,10,11,12,13]. These disparities are not only clinically relevant but also raise important concerns about equity in healthcare delivery and the consistent application of evidence-based medicine. Understanding and addressing these differences is essential for improving patient outcomes and ensuring high-quality care for all individuals, regardless of sex.

Studies have shown that women with PE more commonly present with dyspnea and syncope, whereas men more frequently report chest pain and hemoptysis [5,7,14]. Women are typically older at diagnosis and have more hormone-related risk factors, as well as comorbidities like hypertension and obesity [7,15]. Although general management strategies are similar [7,15], men more often undergo catheter-based interventions, whereas women more frequently receive thrombolysis but are less likely to receive mechanical support [11]. These discrepancies may contribute to outcome differences: women appear to have a higher risk of in-hospital mortality and major bleeding events [12,16], though some studies suggest lower 30-day all-cause mortality compared to men [8,9,10,11]. These complex, sometimes conflicting findings underscore the need for a more equitable clinical approach, as emphasized by the American Heart Association [15].

In Switzerland, a recent nationwide epidemiologic study found no major sex-based differences in trends related to incidence, mortality, or hospital stay length over a 20-year period [2]. However, this analysis did not include detailed patient-level data regarding clinical presentation, comorbidities, diagnostics, or adherence to management guidelines, elements that are essential to understanding disparities in real-world practice.

To address this knowledge gap, we conducted a retrospective analysis of patients diagnosed with PE with the aim of investigating sex-based differences in clinical presentation, diagnostic workup, treatment strategies, and outcomes. By analyzing this cohort in detail, we aim to identify potential sex-based disparities and establish a foundation for developing a more equitable and personalized approach to the clinical management of PE.

## 2. Materials and Methods

### 2.1. Design and Setting

A 2-year retrospective cohort observational single-center study was conducted at the Cantonal Hospital Baselland (KSBL), a public teaching hospital in Switzerland that serves approximately 300,000 residents [17]. In 2023, the hospital registered around 23,000 inpatient admissions [18].

### 2.2. Study Population

**Figure 1:**
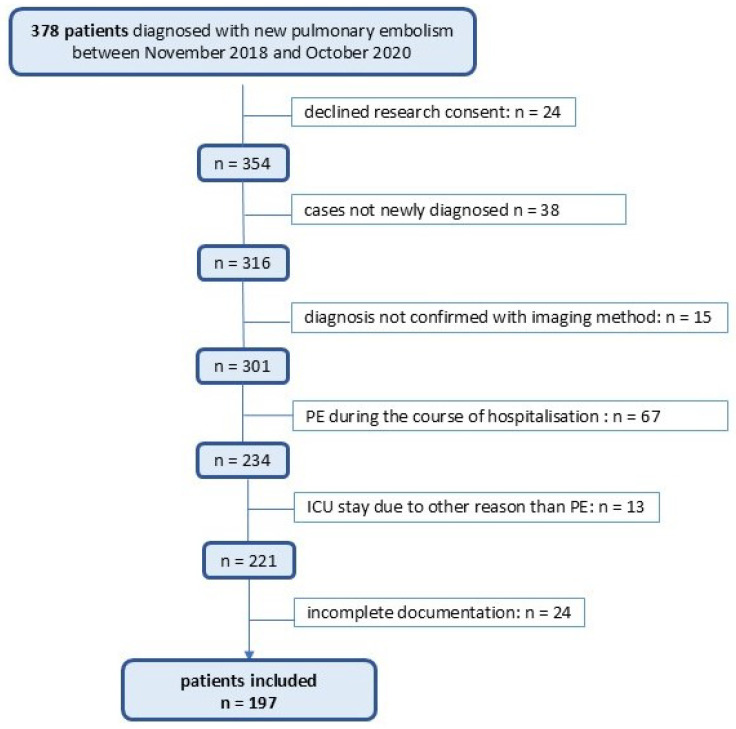
Flowchart of the screening and enrolment of patients.

We included all adult patients (≥18 years) who were hospitalized with a main diagnosis of acute PE between 1 January 2018 and 31 December 2020, who provided general research consent or whose data use was permitted under the ethics committee’s consent exception (Art. 34 HFG). Patients who actively denied general research consent were excluded. Cases were identified using the ICD-codes I26.0 (pulmonary embolism with acute cor pulmonale) and I26.9 (pulmonary embolism without acute cor pulmonale). Among 378 initially identified cases, 197 patients met all inclusion criteria and were included in the final analysis. The flowchart outlining the patient inclusion criteria is presented in Figure 1. The STROBE Statement—checklist of items that should be included in reports of observational studies is presented in Supplementary Table S1.

### 2.3. Data Collection and Analysis

This study involved a secondary analysis of data obtained from a previously published internal audit [19]. Patient data were manually extracted from the hospital information system and the electronic patient records. Extracted data included sociodemographic characteristics, relevant comorbidities, clinical presentation, diagnostic and treatment modalities, discharge management, and key outcomes such as length of stay, ICU transfer, and mortality.

### 2.4. Statistical Analysis

All continuous variables were assessed for normality using histograms and the Shapiro–Wilk test. As none of the continuous variables followed a normal distribution, group comparisons were conducted using the Mann–Whitney U test. Categorical variables were analyzed using the Chi-square test or Fisher’s exact test, as appropriate. In addition to univariate analyses, we conducted a multivariable zero-truncated negative binomial regression to assess the association between sex and length of hospital stay (LOHS), adjusting for relevant covariates. No imputation was performed for missing data; analyses were based on available-case data only. A two-sided *p*-value < 0.05 was considered statistically significant. All analyses were performed using STATA software, version 18 (StataCorp, College Station, TX, USA). Given the retrospective nature of the study, no formal sample size calculation was performed a priori. However, a post-hoc power analysis was conducted based on the observed mortality rate of 23.5% in the study cohort. This analysis aimed to assess the statistical power to detect differences in key outcomes with the available sample size. Details of the post-hoc power calculation, including assumptions and statistical parameters, are provided in the Appendix (see Appendix A Table A3).

## 3. Results

### 3.1. Patient Characteristics

A total of 197 patients with PE were included, with a sex distribution of 107 (54%) females and 90 (46%) males. Patient characteristics are summarized in Table 1. There were no significant differences in sociodemographic characteristics. The median age was 74 years in the overall population (median 74 [64–85] in women vs. 72 [55–80] in men, *p* = 0.130), and the median BMI was 27.5 kg/m^2^ (27 in women vs. 28 in men, *p* = 0.587). Women were more often admitted by paramedics than men (42% vs. 24%, *p* = 0.009). The most common comorbidities in the overall cohort were hypertension (50%), cardiovascular disease (35%), and mental health conditions (25%). Women had a significantly higher prevalence of mental illness (31% vs. 18%, *p* = 0.035). Regarding pre-existing medication, 81% of patients were on chronic treatment at the time of admission. Hormonal therapy was reported significantly more often in women (35% vs. 9%, *p* < 0.001), while no sex differences were found in the use of anticoagulants. At presentation, increased dyspnea (67%), chest pain (43%), and cough (27%) were the most frequently reported symptoms. Men more often reported abdominal pain (11% vs. 4%, *p* = 0.045), while other symptoms, such as pleuritic pain, hemoptysis, or nausea, did not differ significantly by sex.

### 3.2. Anamnesis and Diagnostics

As summarized in Table 2, several aspects of anamnesis and documentation differed by sex. Family history of PE was more frequently documented in men than in women (51% vs. 36%, *p* = 0.038), as were B symptoms such as fever, night sweats, weight loss (31% vs. 12%, *p* = 0.001), and smoking status (67% vs. 48%, *p* = 0.007). However, rates of current or former smoking did not significantly differ. On clinical examination, tachycardia (heart rate >100 bpm) was more frequently observed in women (38% vs. 23%, *p* = 0.024), as was calf pressure pain (28% vs. 11%, *p* = 0.031). Other vital signs, such as respiratory rate and fever, showed no significant sex-based differences. Compression ultrasonography of the legs was performed in 57% of patients, and deep vein thrombosis (DVT) was diagnosed in 73% of those examined. DVT was more frequently detected in women than in men (82% vs. 64%, *p* = 0.035). Diagnostic procedures, including ECG, D-dimer testing, and CT pulmonary angiography, were performed similarly in both sexes. Leg ultrasound was conducted in 57% of patients overall, with no sex difference in frequency. However, women were more likely to receive ultrasound based on clinical symptoms (32% vs. 19%, *p* = 0.009). The rate of inappropriate D-dimer testing—either unnecessary measurement (30%) or omission when indicated (11%)—was similar between groups. Guideline adherence for PE diagnostic workup was suboptimal in general but balanced across sexes (58% in women vs. 57% in men, *p* = 0.857).

### 3.3. Etiology and Risk Classification

As shown in Table 3, there were no significant sex differences in the etiology, location, or extent of PE. The majority of events were idiopathic (61%) and bilateral (64%) in both sexes. A provoked PE was identified in 39% of patients, with no sex-specific variation (44% in women vs. 32% in men, *p* = 0.093).

Regarding PE localization and extension, bilateral emboli were most common (64%), followed by segmental (44%) and paracentral (28%) involvement, with no sex-related differences.

### 3.4. Management and Follow-Up

As shown in Table 4, most aspects of treatment and management were comparable between sexes, with some notable exceptions. Women were significantly more likely to receive thrombolytic (lysis) therapy upon admission than men (10% vs. 2%, *p* = 0.023). The median time from admission to anticoagulation was slightly shorter in women (187 min) than in men (198 min), though this difference did not reach statistical significance (*p* = 0.066). The use of oral anticoagulation (OAC), initial heparin therapy (both UFH and LMWH), and supportive measures such as physiotherapy, inhalation therapy, and compression stockings did not differ significantly between groups. The majority of patients (94%) received OAC, and 61% were treated with initial heparin.

Length of hospital stay was modestly but significantly longer in women (median 6 days vs. 5 days, *p* = 0.044), although hospital costs per night were similar across sexes. In-hospital mortality was low overall (5%) and did not significantly differ between women (7%) and men (3%, *p* = 0.307). However, significant discrepancies have been identified with regard to the patients’ place of residence following their discharge. The majority of men (97%) were discharged to home. Although the majority of women were also discharged to home (81%), women were significantly more likely to be transferred to a rehabilitation facility or another hospital. To further investigate the observed sex difference in LOS, we performed a zero-truncated negative binomial regression, adjusting initially for age and PESI score calculation. In this model, female sex remained significantly associated with a longer hospital stay (IRR: 1.23, 95% CI: 1.01–1.49, *p* = 0.040). However, when rehabilitation was added as a covariate, the association between sex and LOS was no longer statistically significant (IRR: 1.12, *p* = 0.233). In contrast, rehabilitation was strongly associated with increased LOHS (IRR: 1.72, 95% CI: 1.28–2.32, *p* < 0.001), suggesting that post-acute care planning may partially mediate the sex-based difference in hospitalization duration. These results highlight the importance of considering discharge pathways and rehabilitation needs in future research on sex-specific outcomes in PE. Full regression results are presented in Appendix A Table A1 and Table A2.

Despite this, planned follow-up was comparable (70% in women vs. 80% in men, *p* = 0.100), as was the rate of rehospitalization within six months (21% in women vs. 22% in men, *p* = 0.889). Cancer screening after PE was initiated in half of the cohort, but men more frequently underwent laboratory-based cancer screening (46% vs. 16%, *p* = 0.001), whereas other imaging-based modalities (e.g., abdominal ultrasound or CT) were used similarly between sexes. This discrepancy in laboratory work-up was not explained by clinical differences between groups and may reflect practice variation or unconscious bias rather than guideline-driven or evidence-based differences in cancer risk. This observation warrants further investigation to better understand the underlying causes and ensure equitable diagnostic practices.

## 4. Discussion

### 4.1. Clinical Characteristics

In our cohort of patients hospitalized with PE, no major differences in demographic or clinical baseline characteristics between sexes were observed, though a slightly higher proportion of admissions were women.

Women were twice as likely to be admitted via ambulance. This may reflect a more acute presentation in women, often characterized by symptoms such as dyspnea and tachycardia. The literature supports the notion that women present with more severe clinical signs at admission, including vital sign abnormalities [20]. Another plausible explanation relates to social factors: women, especially older ones, are more likely to live alone due to longer life expectancy, increasing reliance on emergency services [21,22].

Our data show a trend towards the usual gender distribution of comorbidities, with women less likely to have a history of smoking, less likely to have cardiovascular disease or dyslipidemia, but significantly more likely to have mental illness [7,14].

A comparative study conducted in the United States revealed that women presented at a higher age, yet the average age of patients in the US cohort was 10 years younger than in our study [14]. Some of the literature indicates that women presented more frequently with dyspnea and less frequently with hemoptysis and chest pain [7,14]. These findings align with the observed trend in our data. Findings from the SERIOUS-PE study identified that women were more likely to have a provoked PE due to varicose veins, hormonal therapy, immobility resulting from hospitalization, and depression (immobility) [7]. The likelihood of an unprovoked PE was described to be higher in men. We did not find any significant differences in our results for sex differences in provoked or unprovoked PE.

The higher use of hormonal therapy in women is consistent with their increased risk for hypercoagulability [23,24]. The risk assessment conducted at the time of admission revealed that the number of high-risk cases among women and men was comparable. In order to validate the initial assessment and ascertain whether the assessment favored one sex, we investigated the number of patients with no high assessment who were subsequently admitted to the IMC/ICU (or who died). A significant difference was not found; however, an observable trend indicated that men were more likely to receive an under-assessed score.

### 4.2. Anamnesis and Diagnostics

A discrepancy was observed in the scope of the anamnesis, with male subjects being asked more frequently about their smoking habits, b-symptoms, and family history. The authors were unable to identify a rationale for this disparity in the anamnesis. It is possible that treating physicians consider men more likely to smoke, whereas women are more likely to have a hormonal etiology. This discrepancy in medical-history-taking is concerning, especially given its relevance for determining PE etiology, and warrants further investigation. Prior research indicates that women often experience longer delays between symptom onset and diagnosis, partly due to differences in how anamnesis is conducted [25]. All other diagnostic procedures were applied equally across sexes.

Differences in the documentation of key anamnestic variables are clinically significant, as they are crucial for assessing cancer risk and guiding etiologic evaluation of PE. Under-documentation in women may result in missed or delayed identification of underlying conditions, including occult malignancy. While adherence to diagnostic guidelines was similar, inconsistent history-taking may reflect unconscious bias or differing clinical assumptions, contributing to inequities in care. These findings highlight the need for standardized, equitable clinical assessments, especially in emergency settings.

### 4.3. Etiology and Risk Classification

Our findings indicate that while sex disparities were evident in some aspects of clinical care and presentation, no significant differences were observed in the etiology, localization, or anatomical extent of PE.

Among patients diagnosed with PE, the majority of cases were idiopathic and bilateral, consistent with the existing literature that reports idiopathic PE as a common presentation in both sexes. Although provoked PE appeared slightly more frequently in women (44%) than in men (32%), this difference did not reach statistical significance. Similarly, the distribution of emboli—whether localized to the right, left, or bilaterally—was comparable between sexes, as was the extent of involvement, with segmental and paracentral emboli being most common.

The absence of significant sex differences in PE localization or extent aligns with previous studies, suggesting that while biological sex may influence thromboembolic risk profiles, it does not necessarily alter the physical characteristics of the embolism. However, our broader dataset indicated that women with PE were more likely to present with tachycardia, concurrent deep vein thrombosis, and longer hospital stays, and received thrombolysis more promptly pointing to potential sex-related variations in disease severity or healthcare response rather than disease anatomy.

### 4.4. Management and Follow-Up

Most patients received an oral anticoagulant in addition to heparin. Although women had a shorter median time from admission to initiation of anticoagulation compared to men (187 min vs. 198 min), this difference did not reach statistical significance. This trend may relate to the higher proportion of women admitted via ambulance, potentially reflecting earlier triage or prioritization in emergency care. However, since the time from admission to CT was comparable across sexes and only a minority of patients received anticoagulant loading before imaging, it is unlikely that mode of admission alone accounts for the observed trend. These findings suggest that differences in the timing of anticoagulation may reflect case-level variation rather than systematic disparities in care.

Lysis therapy was initiated in only a small number of patients (7%), and it was observed that a significantly higher proportion of women received lysis therapy. Thrombolysis was administered based on ESC guideline recommendations, typically in cases of hemodynamic instability, right-ventricular dysfunction on imaging, or significantly elevated cardiac biomarkers (e.g., troponin or NT-proBNP). Even though women received a more rapid and thorough initial treatment, we did not find any differences in outcome that would reflect these differences in the initial treatment regimen. Despite the more aggressive therapy, women did not have a better survival rate, lower rehospitalization rate, or shorter duration of hospital stay. No differences were observed with regard to in-hospital mortality or rehospitalization. One possible explanation for these findings is that women presented with more severe embolic events, which may have resulted in a more aggressive therapeutic approach and longer hospital stays. Other reasons for differences in reported hospital stay without significant differences in outcomes may be related to factors not captured in our study, such as social circumstances, access to post-discharge care, or psychological well-being.

The available literature is not entirely consistent regarding differences in management or outcomes. A recent study from Israel found no differences in risk stratification, diagnostics performed, therapies, or outcomes [14]. A review concluded that women are more likely to be suspected of having pulmonary embolism. As the overall incidence of PE is similar in women and men, there appears to be an overdiagnosis in women, partly explained by sex differences in d-dimer levels [20]. The literature is conflicting on the association between sex and likelihood of thrombolysis, with women tending to receive less thrombolysis [20,26,27]. We found less comprehensive anamnesis for women, but no other difference in diagnoses.

We observed a clear sex difference in discharge destination: men were more likely to return home, while women were more frequently transferred to rehabilitation centers or other care facilities. This likely reflects the social and health dynamics of older populations. Due to their longer life expectancy, older women are more often widowed or living alone, making a return home less feasible without support. In contrast, men are more likely to have a partner at home who can assist with care. While some studies suggest that women may be less motivated or adherent to rehabilitation programs, the literature on sex differences in rehabilitation engagement remains inconclusive [28,29]. Interestingly, the length of stay after discharge had no effect on the rehospitalization rate [30].

Given the comparable incidence of unprovoked pulmonary embolism between male and female patients, similar rates of cancer screening would be expected. While overall screening rates did not differ significantly, a notable sex-based difference was observed in the use of laboratory-based cancer diagnostics, which were more frequently conducted in men. This discrepancy merits further investigation, particularly in light of current clinical guidelines that recommend baseline malignancy screening—including laboratory testing—for all patients presenting with unprovoked PE [31,32]. The observed difference may reflect sex-specific variations in cancer types and the corresponding diagnostic approaches. For example, prostate cancer in men can be detected through blood tests, whereas breast cancer in women typically requires imaging such as mammography. The overall low use of abdominal CT imaging in both sexes is consistent with recent evidence, suggesting limited clinical utility of routine extended cancer screening in the context of unprovoked PE [33].

### 4.5. Strengths and Limitations

Despite increasing research examining sex differences in clinical presentation and patient characteristics, few studies have systematically examined disparities in management, where multiple influencing factors intersect. Our analysis highlights differences not only in presentation but also in management, therapy response, and adherence. By addressing these aspects comprehensively, we can identify potential discrepancies in outcomes and explore their underlying causes. However, some limitations of our study must be acknowledged.

Furthermore, the retrospective design limited our ability to capture certain clinically relevant variables, such as detailed smoking history or psychosocial context, which may contribute to observed differences in presentation, management, or outcomes. We were also unable to evaluate the degree of control of relevant comorbidities, such as hypertension or diabetes, as key clinical parameters (e.g., HbA1c, insulin dosage, lipid values) were not systematically documented in a retrievable format. Given that all data were manually extracted and validated by physicians to ensure high accuracy, additional retrospective collection was not feasible.

It is also important to note that we did not control for confounders, as we primarily employed univariate analyses to explore group differences in this exploratory study. Although logistic regression could help identify independent associations, we refrained from using it due to the low number of outcome events (e.g., thrombolysis, in-hospital mortality), which would result in underpowered and potentially unstable models. The study’s descriptive aim was to provide an initial overview of sex-based differences in a real-world hospital cohort, rather than to establish causal relationships. Future research should consider applying multivariable models to adjust for potential confounders and interactions. In particular, variables such as social status and domestic situation, which may influence admission type, length of stay, or discharge destination, should be incorporated in future studies to better understand the underlying drivers of the observed disparities.

The retrospective and single-center design of this study, combined with the relatively small sample size, limit the statistical power to detect smaller effects and reduce the generalizability of the findings to broader populations. Larger multi-center studies are needed to validate our observations.”

## 5. Conclusions

In this retrospective study of patients hospitalized with acute PE, we identified notable sex-based differences in clinical presentation, diagnostic workup, and discharge disposition. Women were more likely to present with tachycardia, receive thrombolysis, and be discharged to rehabilitation facilities, while men were more frequently discharged home. Documentation of relevant clinical history (e.g., smoking, B symptoms) was more complete in men, suggesting possible bias or differences in clinical focus.

Importantly, no sex-based differences were found in key clinical outcomes such as in-hospital mortality or 6-month rehospitalization. These findings underline the importance of standardizing history-taking and discharge planning to ensure equitable care. Further multicenter studies using multivariable analysis are needed to better understand the drivers of observed disparities and guide more personalized and equitable PE management.

## Figures and Tables

**Table 1:** Patient characteristics of people hospitalized with PE.

Patient Characteristics	n (%)	Female	Male	Missing Data	*p*-Value
Patients overall	N = 197	107 (54)	90 (46)		
Demographics				0	
Age, years (median [IQR]) (*range*)	74 [62–83] (19–96)	74 [64–85] (22–96)	72 [55–80] (19–94)		0.130
Resident at a care facility	32 (16)	21 (20)	11 (12)		0.160
BMI kg/m^2^	176 (89)	95 (89)	81 (90)	21 (11)	
(median [IQR])	27.5 [24–32]	27 [24–32]	28 [25–31]		0.587
Admission				0	
Paramedics	67 (34)	45 (42)	22 (24)		0.009
Comorbidities				0	
Cardiovascular disease	69 (35)	31 (29)	38 (42)		0.052
Diabetes mellitus	31 (16)	16 (15)	15 (17)		0.742
Dyslipidemia	38 (19)	17 (16)	21 (23)		0.187
Hypertension	98 (50)	50 (47)	48 (53)		0.356
Chronic lung disease	35 (18)	15 (14)	20 (22)		0.133
Mental disease	49 (25)	33 (31)	16 (18)		0.035
Varicose or CVI	29 (15)	12 (11)	17 (19)		0.130
Medication before admission	160 (81)	91 (85)	69 (77)	0	0.134
Anticoagulation	9 (5)	6 (6)	3 (3)		0.446
Hormones	45 (23)	37 (35)	8 (9)		0.000
Symptoms				0	
increased dyspnea	132 (67)	76 (71)	56 (62)		0.190
Inspiration pain	56 (28)	26 (24)	30 (33)		0.161
Chest pain	85 (43)	45 (42)	40 (44)		0.736
Back pain	20 (10)	12 (11)	8 (9)		0.590
Abdominal pain	14 (7)	4 (4)	10 (11)		0.045
Cough	54 (27)	24 (22)	30 (33)		0.087
Hemoptysis	8 (4)	2 (2)	6 (7)		0.089
Nausea/Vomiting	22 (11)	16 (15)	6 (7)		0.066
Weakness/tiredness	40 (20)	24 (22)	16 (18)		0.419

**Table 2:** Anamnesis, clinical examination, diagnostics and documentation upon admission.

Procedure/Documentation	Performed, n (%)	Female	Male	*p*-Value
Patients overall	N = 197	107 (54)	90 (46)	
Anamnesis and documentation				
Family history asked	85 (43)	39 (36)	46 (51)	0.038
b-symptoms *	41 (21)	13 (12)	28 (31)	0.001
Smoking status	111 (56)	51 (48)	60 (67)	0.007
Current smoker	28 (25)	10 (20)	18 (30)	0.209
Former smoker	35 (33)	12 (25)	23 (40)	0.096
DVT symptoms checked	105 (53)	57 (53)	48 (53)	0.993
Clinical examination				
Tachypnea (>20/min)	77 (39)	47 (44)	30 (33)	0.086
Fever (≥38.5 °C)	10 (5)	6 (6)	4 (4)	0.711
Tachycardia (>100 bpm)	62 (31)	41 (38)	21 (23)	0.024
Pressure pain calf	20 (20)	15 (28)	5 (11)	0.031
Additional Risk factors				
Previous VTE	47 (24)	22 (21)	25 (28)	0.236
Previous DVT	35 (18)	15 (14)	20 (22)	0.133
Previous PE	21 (11)	9 (8)	12 (13)	0.265
Immobility/recent surgery	52 (26)	32 (30)	20 (22)	0.223
History of cancer	33 (17)	17 (16)	16 (18)	0.723
Active cancer	12 (6)	7 (7)	5 (6)	0.773
Family history for PE	85 (43)	39 (36)	46 (51)	
Positive	28 (33)	12 (31)	16 (35)	0.695a
*Leg Sonography*	113 (57)	60 (56)	53 (59)	
*DVT Found*	*83 (73)*	*49 (82)*	*34 (64)*	*0.035*
Risk assessment at admission	189 (96)	99 (93)	90 (100)	
Intermediate high/High risk	52 (28)	29 (29)	23 (26)	0.566
IMC/ICU despite no high risk	29 (15)	11 (11)	18 (20)	0.090
Diagnostics				
Respiratory rate	169 (86)	91 (85)	78 (87)	0.746
ECG	193 (98)	105 (98)	88 (98)	0.861
ECG analyzed	146 (74)	81 (76)	65 (72)	0.597
D-dimers	138 (70)	73 (68)	65 (72)	0.542
D-dimers unnecessarily measured	60 (30)	34 (32)	26 (29)	0.661
D-dimers falsely not measured	21 (11)	10 (9)	11 (12)	0.515
Sonography leg	113 (57)	60 (56)	53 (59)	0.691
Sono based on symptoms	51(26)	34 (32)	17 (19)	0.009
Sono despite not examined	43 (22)	20 (19)	23 (26)	0.272
No Sono despite symptoms	10 (5)	5 (5)	5 (6)	0.686
X Ray chest	46 (23)	21 (20)	25 (28)	0.178
CT pulmonary angiography	189 (96)	102 (95)	87 (97)	0.635
Diagnostics according to guidelines	113 (57)	62 (58)	51 (57)	0.857

DVT: Deep vein thrombosis; ECG: electrocardiogram; CT: computerized tomography, bpm: Beats per minute, VTE: Venous thromboembolism, IMC: Intermediate Care Unit, ICU: Intensive Care Unit, ECG: Electrocardiogram, CT: Computed Tomography, * B-symptoms were defined as the presence of at least one of the following systemic symptoms, as documented in the patient record: Unexplained fever (>38 °C), Unintentional weight loss (>10% of body weight over 6 months), Night sweats.

**Table 3:** Etiology and risk classification of people hospitalized with PE.

Etiology and Risk Classification	n (%)	Female	Male	*p*-Value
Classification of the PE				
Provoked	76 (39)	47 (44)	29 (32)	0.093
Idiopathic	121 (61)	60 (56)	61 (68)	0.093
Localization in the lung				
Right	50 (25)	29 (27)	21 (23)	0.498
Left	17 (9)	10 (9)	7 (8)	0.669
Bilateral	126 (64)	64 (60)	62 (69)	0.237
Maximum extension of the PE				
Central	26 (13)	13 (12)	13 (14)	0.669
Paracentral	55 (28)	31 (29)	24 (27)	0.663
Segmental	86 (44)	48 (45)	38 (42)	0.630

**Table 4:** Therapy, management, follow-up, and outcome. Data presented as n (%) if not otherwise stated.

Management/Follow Up	n (%)	Female	Male	Missing	*p*-Value
Patients overall	N = 197	107 (54)	90 (46)		
Anticoagulant treatment				0	
Loading before CT	31 (16)	18 (17)	13 (14)		0.648
Lysis therapy	13 (7)	11 (10)	2 (2)		0.023
OAC	186 (94)	100 (94)	86 (96)		0.523
Initial heparin	120 (61)	65 (61)	55 (61)		0.958
UFH	47 (39)	30 (46)	17 (31)		0.133
LMWH	88 (73)	47 (72)	41 (75)		0.819
Non-anticoagulant treatment					
Physiotherapy	114 (58)	65 (61)	49 (54)	0	0.372
New inhalation therapy	32 (16)	15 (14)	17 (19)	0	0.883
New compression stockings	44 (22)	25 (23)	19 (21)	0	0.705
Length of stay				0	
days (median [IQR])	5 (4.8) [1–32]	6 (4.8) [1–32]	5 (3.7) [1–24]		0.044
Cost per night	181 (92)	100 (93)	81 (90)	16 (8)	
CHF mean (SD)	2900 (1304)	2897 (1365)	2904 (1233)		0.970
Hospitalization				0	
hospitalized, normal ward	137 (70)	78 (73)	59 (66)		0.342
hospitalized, IMC	23 (12)	10 (9)	13 (14)		0.267
hospitalized, ICU	37 (19)	19 (18)	18 (20)		0.688
Time admission to CT	164 (83)	87 (81)	77 (86)	33 (17)	
min (median [IQR])	121 [90–211]	121 [86–195]	119 [96–222]		0.370
Time admission to anticoagulation	177 (90)	98 (92)	79 (88)	20 (10)	
min (median [IQR])	193 [145–177]	187 [138–249]	198 [150–329]		0.066
Mortality				0	
In hospital death	10 (5)	7 (7)	3 (3)		0.307
Patients overall	N = 187	100 (53)	87 (47)		
Residence after discharge					0.005
Home	165 (88)	81 (81)	84 (97)		
Reha	14 (8)	11 (11)	3 (3)		
Other Hospital	8 (4)	8 (8)	0		
Aftercare					
Cancer search	93 (50)	45 (45)	48 (55)		0.114
Cancer lab	29 (31)	7 (16)	22 (46)		0.001
Sono abdomen	34 (37)	13 (29)	21 (44)		0.098
CT abdomen	15 (16)	8 (18)	7 (15)		0.684
Follow up planned or advised	140 (75)	70 (70)	70 (80)		0.100
Rehospitalization KSBL					
<6 months after discharge	40 (21)	21 (21)	19 (22)		0.889

CT: computerized tomography; OAC: oral anticoagulant; UFH: unfractionated heparin; LMWH: low-molecular-weight heparin; IQR: interquartile range; CHF: Confoederatio Helvetica Franc; SD: standard deviation; Sono: Ultrasound; KSBL: Kantonsspital Baselland Liestal.

## Data Availability

All data generated during this study were analyzed and the results were included in this article. The data presented in this study are available on reasonable request from the corresponding author. The data are not publicly available due to restrictions on data privacy.

## Supplementary Materials

The following supporting information can be downloaded at: https://www.mdpi.com/article/10.3390/jcm14155287/s1, Table S1: STROBE Statement—checklist of items that should be included in reports of observational studies.

## Appendix A

**Table A1:** Zero-Truncated Negative Binomial Regression of Length of Stay Adjusted for Sex, Age, and PESI Score.

Variable	IRR	*p*-Value	95% Confidence Interval
Sex (female)	1.23	0.040	1.01–1.49
PESI score	0.98	0.871	0.77–1.25
Age at entry	1.01	<0.001	1.006–1.019

**Table A2:** Zero-Truncated Negative Binomial Regression of Length of Stay Including Rehabilitation.

Variable	IRR	*p*-Value	95% Confidence Interval
Sex (female vs. male)	1.12	0.233	0.931–1.343
Age at admission (years)	1.012	<0.001	1.006–1.018
PESI score	0.975	0.827	0.778–1.222
Rehabilitation	1.72	<0.001	1.278–2.322

**Table A3:** Post-hoc Power Analysis for Mortality by Sex.

Parameter	Value
Difference in mortality rates (female 7%—male 3%)	4%
Sample size Females (n)	107
Sample size males (n)	90
Significance level (alpha)	0.05
Estimated power (1—β)	23.5%

Power was calculated using Stata command: power twoproportions 0.07 0.03, n1 (107) n2 (90) alpha (0.05).
