# Supplementary material for: Sex-Based Differences in Clinical Presentation, Management, and Outcomes in Patients Hospitalized with Pulmonary Embolism: A Retrospective Cohort Study
Source: J Clin Med. 2025 Jul 26;14(15):5287. doi: 10.3390/jcm14155287 (PMC12346922; doi:10.3390/jcm14155287)
Supplement: Supplementary file 1 [file jcm-14-05287-s001.zip › jcm-3738865-supplementary.pdf]

Table S1. STROBE Statement—checklist of items that should be included in reports of observational studies.

	Item No	Recommendation	Addressed in Manuscript
**Title and abstract**	1	(*a*) Indicate the study's design with a commonly used term in the title or the abstract	✓ Title: "A Retrospective Cohort Study"
		(*b*) Provide in the abstract an informative and balanced summary of what was done and what was found	✓ Structured abstract (page 1) includes background, methods, results, and conclusions
**Introduction**			
Background/rationale	2	Explain the scientific background and rationale for the investigation being reported	✓ Introduction (pages 1–2)
Objectives	3	State specific objectives, including any prespecified hypotheses	✓ Last paragraph of Introduction (page 2)
**Methods**			
Study design	4	Present key elements of study design early in the paper	✓ Materials and Methods" section begins with design description (page 2)
Setting	5	Describe the setting, locations, and relevant dates, including periods of recruitment, exposure, follow-up, and data collection	✓ Setting: KSBL; Dates: Jan 2018–Dec 2020 (page 2)
Participants	6	(*a*) *Cohort study*—Give the eligibility criteria, and the sources and methods of selection of participants. Describe methods of follow-up.<br>*Case-control study*—Give the eligibility criteria, and the sources and methods of case ascertainment and control selection. Give the rationale for the choice of cases and controls.<br>*Cross-sectional study*—Give the eligibility criteria, and the sources and methods of selection of participants	✓ Page 2–3, inclusion/exclusion criteria and flowchart reference
		(*b*) *Cohort study*—For matched studies, give matching criteria and number of exposed and unexposed<br>*Case-control study*—For matched studies, give matching criteria and the number of controls per case	Not applicable
Variables	7	Clearly define all outcomes, exposures, predictors, potential confounders, and effect modifiers. Give diagnostic criteria, if applicable	✓ Clearly defined in Methods and Tables 1–4
Data sources/	8*	For each variable of interest, give sources	✓ Manual data extraction from EMR;

measurement		of data and details of methods of assessment (measurement). Describe comparability of assessment methods if there is more than one group	described in Methods
Bias	9	Describe any efforts to address potential sources of bias	✓ Page 6 (Discussion – limitations)
Study size	10	Explain how the study size was arrived at	✓ No a priori sample size; post-hoc power analysis in Appendix A3
Quantitative variables	11	Explain how quantitative variables were handled in the analyses. If applicable, describe which groupings were chosen and why	✓ Explained in Statistical Analysis section (page 3)
Statistical methods	12	(*a*) Describe all statistical methods, including those used to control for confounding	✓ Univariate tests, multivariable negative binomial, post-hoc power analysis (page 3, Appendix)
		(*b*) Describe any methods used to examine subgroups and interactions	Not conducted
		(*c*) Explain how missing data were addressed	✓ Missing values clearly reported in Tables, no imputation was performed for missing data as explain in methods.
		(*d*) *Cohort study*—If applicable, explain how loss to follow-up was addressed<br>*Case-control study*—If applicable, explain how matching of cases and controls was addressed.<br>*Cross-sectional study*—If applicable, describe analytical methods taking account of sampling strategy	Not applicable (retrospective design)
		(*e*) Describe any sensitivity analyses	Not conducted

Continued on next page

**Results**			
Participants	13*	(a) Report numbers of individuals at each stage of study—eg numbers potentially eligible, examined for eligibility, confirmed eligible, included in the study, completing follow-up, and analysed	✓ Figure 1: flow diagram
		(b) Give reasons for non-participation at each stage	✓ Explained in the methods
		(c) Consider use of a flow diagram	✓ Yes, Figure 1
Descriptive data	14*	(a) Give characteristics of study participants (eg demographic, clinical, social) and information on exposures and potential confounders	✓ Table 1 (demographics, comorbidities)
		(b) Indicate number of participants with missing data for each variable of interest	✓ Yes, in each table
		(c) *Cohort study*—Summarise follow-up time (e.g., average and total amount)	Not applicable
Outcome data	15*	*Cohort study*—Report numbers of outcome events or summary measures over time	✓ Presented in Tables 2–4
		Case-control study—Report numbers in each exposure category, or summary measures of exposure	Not applicable
		Cross-sectional study—Report numbers of outcome events or summary measures	Not applicable
Main results	16	(*a*) Give unadjusted estimates and, if applicable, confounder-adjusted estimates and their precision (e.g., 95% confidence interval). Make clear which confounders were adjusted for and why they were included	✓ p-values reported in all tables, IRRs and CI reported in Tables A1 and A2
		(*b*) Report category boundaries when continuous variables were categorized	✓ Yes median and IQR
		(*c*) If relevant, consider translating estimates of relative risk into absolute risk for a meaningful time period	Not applicable (no relative risk reported)
Other analyses	17	Report other analyses done—eg analyses of subgroups and interactions, and sensitivity analyses	✓ Regression on LHOS (Appendix A1–A2), power post-hoc analysis
**Discussion**			
Key results	18	Summarise key results with reference to study objectives	✓ Pages 6–9
Limitations	19	Discuss limitations of the study, taking into account sources of potential bias or imprecision. Discuss both direction and magnitude of any potential bias	✓ Page 9–10
Interpretation	20	Give a cautious overall interpretation of results considering objectives, limitations, multiplicity of analyses, results from similar studies, and other	✓ Pages 6–10

		relevant evidence	
Generalisability	21	Discuss the generalisability (external validity) of the study results	✓ Page 10 (single-center, caution advised)
**Other information**			
Funding	22	Give the source of funding and the role of the funders for the present study and, if applicable, for the original study on which the present article is based	✓ Page 11: "no external funding" COI disclosed
